# Price variation among different brands of anticancer medicines available in hospital pharmacies of Nepal

**DOI:** 10.1186/s40545-020-0203-0

**Published:** 2020-04-02

**Authors:** Sunil Shrestha, Ramesh Sharma Poudel, Bhuvan KC, Bhupendra Kumar Poudel, Binaya Sapkota, Sabina Sharma, Anil Khadka

**Affiliations:** 1 Department of Pharmacy, Nepal Cancer Hospital and Research Center, Harisiddhi, Lalitpur, Nepal; 2 Department of Pharmaceutical and Health Service, Nepal Health Research and Innovation Foundation, Lalitpur, Nepal; 3 Graduate School of Health, University of Technology Sydney, Sydney, NSW Australia; 4 School of Pharmacy, Monash University Malaysia, Jalan Lagoon Selatan, 47500 Bandar Sunway, Selangor Malaysia; 5 Department of Drug Administration, Bijulibajar, Kathmandu, Nepal; 6 Department of Pharmaceutical Sciences, Nobel College, Affiliated to Pokhara University, Sinamangal, Kathmandu, Nepal; 7 Department of Public Health, Nobel College, Affiliated to Pokhara University, Sinamangal, Kathmandu, Nepal

**Keywords:** Affordability, Anti-cancer medicines, Price analysis, Drug price, Nepal

## Abstract

**Objective:**

To assess the variation in price among different brands of anticancer medicines available in hospital pharmacies at Nepalese cancer hospitals.

**Methods:**

The price of different brands of the same anticancer medicines available in the hospital pharmacies of two cancer hospitals was assessed. Prices of different dosage forms such as a single tablet, capsule and vial were calculated. The difference in the maximum and minimum price of the same drug manufactured by different pharmaceutical industries was determined, and the percentage variation in price was calculated. The prices of medicines (brands) were also compared with the price determined by the government where available.

**Results:**

Price variation was assessed for 31 anticancer medicines belonging to six broad categories. Prices were found to vary maximally among the following medicines, each belonging to separate categories: among alkylating agents, the price of temozolomide 100 mg capsule varied 308%; among antimetabolite agents, the price of pemetrexed 500 mg injection varied 134%; among hormonal drugs, the price of letrozole 2.5 mg tablet varied 200%; among antibody class, the price of trastuzumab 440 mg injection varied 73%; among natural products, the price of irinotecan 100 mg injection varied 590%; and among miscellaneous agents, the price of bortezomib 2 mg injection varied 241%. There was a significant difference in the mean MRP of the alkylating agents with the antimetabolites (*p*-value 0.006) and the monoclonal antibody (*p*-value <.001). Antimetabolites, natural products, hormonal therapy all had significant mean differences in their MRPs with the monoclonal antibodies. (*p*-value <.001) and the monoclonal antibodies had a significant mean difference in the MRP with the miscellaneous agents. (*p*-value <.001).

**Conclusions:**

There was a considerable variation in the price of different brands of anticancer medicines available in the Nepalese market. The Government of Nepal has regulated the prices of some medicines, including anticancer medicine. However, it is not enough as prices of the majority of anticancer medicines are still not regulated. Therefore, further strategies are needed to address the variation in the prices of anticancer medicines available in the Nepalese market.

## Introduction

Globally, cancer is the second leading cause of death and is accountable for a probable 9.6 million deaths in the year 2018 [1]. In a systematic analysis by the Global Burden of Disease Cancer Collaboration in 2016, there were 17.2 million incident cancer cases, 8.9 million deaths, and 213.2 million Disability-Adjusted Life-Years (DALYs) due to cancer worldwide [2]. It is one of the major non-communicable diseases (NCDs) in Nepal [3]. Age-standardized cancer incidence and mortality rates in Nepal have been estimated to be 103.7 and 77.8 per 100,000 populations respectively in 2018 [4]. Cancer is a major social and financial burden to Nepalese society and the healthcare system. Chemotherapy is one of the common treatment modalities for cancer patients [5]. Anti-cancer medicines are usually expensive [6] and cause financial distress to the patients, society, and the country. Studies have reported significant variation in the prices of the same anti-cancer medicines across different countries (up to 388% variation) [7, 8] and within a country (up to 714% variation) [9].

Nepal lacks a comprehensive public healthcare financing system. The government has started a basic Health Insurance Policy with partial financial coverage in 39 districts of Nepal from April 2016 [10]. This partial support has various limitations and it is not readily available to the public [11]. The Government of Nepal (GoN) provides a partial financial subsidy of Nepalese rupees (NRs) 100,000 (Euro 877.19, USD 925.92) for cancer or other major health problems requiring tertiary care, which is a one-off payment. This subsidy covers the price of anticancer medicines and radiotherapy treatment only but does not cover bed charges, laboratory tests, radiological examinations, and others treatment cost. It can be accessed via both the private and government cancer hospitals [12]. However, this financial support for cancer patients is inadequate as it covers a very small fraction of the estimated average treatment cost [13]. The direct average cost of cancer care in the government hospital of Nepal was estimated to be NRs 387,000 [Euro 3076.46, USD 3423.23] which are unaffordable to the Nepalese population [13]. The cost of oncology treatment varies between hospitals with higher costs in private hospitals [14]. Therefore, the majority of Nepalese patients, including cancer patients, rely on out-of-the-pocket expenditure for the treatments of cancer and other major health problems [13, 15, 16].

The variation in the price of the same medicines manufactured by different pharmaceutical companies might have a profound effect on patients’ healthcare expenditure, access to treatment and overall treatment outcome. To address this issue the GoN has published a notice in the Nepal Gazette in August 2015 regarding the control of prices of a few common medicines, including anticancer medicines [17]. Based on this, the Department of Drug Administration (DDA) controls the maximum retail price (MRP) of those 117 pharmaceutical products in the Nepalese market [17]. This price control regulation is effective in controlling the prices of the limited number of pharmaceutical products available in the Nepalese market [18], but the inclusion of a limited number of pharmaceutical products in the price control list may allow pharmaceutical industries to sell other products at a higher cost. Previous studies from Nepal have reported substantial price variation of medication used for the management and treatment of NCDs [18–20]. However, these studies did not include anticancer medicines. Thus, information on the price, price variation, and other cost factors of different anticancer medicines available in the Nepalese market are lacking.

Currently, there are 12 cancer hospitals that provide diagnostic and treatment services for cancer patients in Nepal. Of these 12 hospitals, five of them are government hospitals. Radiation therapy, chemotherapy, and surgery are available treatment options in Nepal for cancer patients. However, all these services are not available in all hospitals; only five hospitals (both private and government) out of the 12 cancer hospitals provide comprehensive cancer treatment services for different types of cancers.

There are more than 70 domestic pharmaceutical companies in Nepal [21] and none currently manufactures anticancer medicines. Nepal has been importing anticancer medicines mainly from India and Bangladesh [21]. Study on the prices of different brands of anticancer medicines is necessary to find out discrepancies in the prices of different brands of the same medicines manufactured by numerous international pharmaceutical industries. The findings of this study will provide evidence to prescribers, patients and regulatory bodies to develop and implement strict policies and strategies aimed at improving affordability and access to anticancer medicines. This study aims to assess the variation in prices of different brands of anticancer medicines available in the two private cancer hospital pharmacies of Nepal.

## Methods

### Study design

A cross-sectional study was conducted from April 2019 to May 2019. Two private cancer hospitals were purposively selected for the study, as they were the major specialized private hospitals for cancer care in Nepal. These cancer hospitals provide comprehensive treatment including, radiation therapy, chemotherapy, and surgery to the cancer patients. The majority of cancer patients come to Kathmandu valley for a treatment, which is also a capital city of Nepal. Both hospitals together cover 25–35% of cancer patients which is an estimated data obtained from respective hospitals.

### Sampling technique

A market basket method, including a total collection of all available anticancer medicines available at the two selected hospital pharmacies, was used for sample collection.

### Inclusion

Individual anticancer medicine having the same dose and dosage forms manufactured by ≥2 different pharmaceutical companies were included, only medicines actually available on date April-May 2019.

### Exclusion criteria

- Brands with no competitor (i.e., just single brand available) and formulations containing a combination of anticancer medicines were excluded.
- Modified-release formulations were not included in price comparison as modified-release formulations were not available at both pharmacies.
- We excluded government hospitals in Nepal as there is a different purchasing system of medicines in government hospitals.

### Data collection

- The research team identified the available anticancer medicines at two hospitals pharmacies of cancer hospital by physically checking all the available medicines.
- MRP i.e. selling prices in NRs were obtained from the drug dispensing software. Patients usually pay the exact amount indicated in MRP. It was again cross-verified by checking the price mentioned in the labeling.
- The procurement of medicines at both hospitals is a direct- and competitive-procurement model. Procurement of medicines at private hospitals depends on lesser cost price or recommendations of doctors or the availability of drugs. Examining the procurement price of anticancer medicines from the private hospital of Nepal is difficult.
- In the case of government hospitals, procurement is mainly based on a tender system. Whatever the price of procurement is, the MRP of the medicines doesn’t change.

### Data analysis

The price denotes the individual medicines MRP in their respective labels. The retail price of each unit of the individual drug with the same strength and dosage form from all manufacturers were calculated. Price of the oral anticancer medicine formulation was calculated for a single tablet or capsule. The price of the injectable medicine was calculated per vial.

The difference in the maximum and minimum price of the individual drug being manufactured by several pharmaceutical industries across different brands was calculated using the following formula: % Price variation = [Price of the brand with the highest price- price of the brand with the lowest price] × 100/Price of the brand with the lowest price.

Anticancer medicines were further categorized into six different therapeutic class i.e., alkylating agents, antimetabolite agents, natural products, hormonal therapy, monoclonal antibodies, and miscellaneous agents [22] and price variation was compared within the category. The MRP indicated in the anticancer medicines label available at two pharmacies (calculated per unit) was compared with the Nepalese government fixed price where available [17]. The available medicines were also compared with the price set by the government. Data management and analysis were performed with the help of MS-Excel 2010 (Microsoft Corporation, Redmond, WA, USA) and SPSS Version 26 (SPSS Inc., Chicago, IL, USA, 2019). Between-group mean difference in the MRP of the anticancer medications was performed with a one-way ANOVA test. The *p*-value < 0.05 was considered statistically significant.

## Results

The variation in the price of 31 anticancer medicines belonging to six categories was assessed (Table 1). The maximum variation was found in irinotecan 100 mg injection (590.30%).

**Table 1:** Price variation among different brands of anticancer medicines available in two private hospital pharmacies of Nepal

Generic Name	Strength	Dosage Form	No. of available brands	Government price (NRs per unit)	Min. Price (NRs per unit)	Max. Price (NRs per unit)	Price Variation (%)	Median Price	IQR
Bendamustine hydrochloride	100 mg	Injection	2	GPNA	11,120.00	12,000.00	7.90	11,560.00	–
Carboplatin	150 mg	Injection	8	1393.87	1184.00	1393.60	17.70	1342.60	179.70
	450 mg	Injection	7	4130.77	3476.8	4028.00	15.90	3920.00	472.50
Cisplatin	10 mg	Injection	5	149.87	100.00	128.00	28.00	113.00	21.20
	50 mg	Injection	6	532.21	512.00	532.20	3.90	520.00	123.50
Cyclophosphamide	1 g	Injection	3	GPNA	115.70	204.80	77,00	176.00	–
	500 mg	Injection	2	105.23	101.80	105.20	3.30	105.20	–
	200 mg	Injection	4	GPNA	51.20	80.00	56.30	66.00	110.20
Oxaliplatin	50 mg	Injection	7	4035.86	3336.50	4035.90	21,00	4000.00	595.90
	100 mg	Injection	7	GPNA	6138.70	7516.60	22.40	7046.70	811.20
Temozolomide	20 mg	Capsule	3	GPNA	607.70	1232.00	102.70	960.00	–
	250 mg	Capsule	6	GPNA	2550.00	6400.00	151.00	4549.00	3181.00
	100 mg	Capsule	5	GPNA	783.30	3193.80	307.70	2521.80	1430.60
Cytosine Arabinoside (Cytarabine)	500 mg	Injection	2	910.74	807.44	807.55	0	794.10	–
	1000 mg	Injection	2	2068.00	1604.80	2068.00	28.90	2068.00	–
	100 mg	Injection	3	421.23	279.39	421.23	50.80	302.80	–
Capecitabine	500 mg	Tablet	7	GPNA	188.80	261.00	38.20	212.60	68.80
Gemcitabine	1400 mg	Injection	2	GPNA	9920.00	13,400.90	35.10	11,660.40	–
	1000 mg	Injection	10	9980.00	7072.00	9980.00	41.10	8626.60	902.60
	200 mg	Injection	8	1706.93	1704.00	1706.90	0.20	1706.90	1.23
Pemetrexed	100 mg	Injection	5	GPNA	5880.00	11,200.00	90.50	6560.00	4220.00
	500 mg	Injection	8	GPNA	20,512.00	48,000.00	134,00	31,040.00	13,960.00
Daunorubicin	20 mg	Injection	2	592.18	592.20	592.20	0	592.17	0
Docetaxel	20 mg	Injection	8	GPNA	3144.00	5600.00	78.10	4948.66	848.90
	80 mg	Injection	9	GPNA	7832.00	19,142.10	144.40	17,760.00	4227.90
	120 mg	Injection	3	GPNA	24,464.00	28,342.60	15.90	24,800.00	-
Doxorubicin	10 mg	Injection	5	328.00	264.00	328.00	24.20	300.8	30.00
	50 mg	Injection	7	1416.28	1280.00	1416.30	10.60	1375.00	121.40
Doxorubicin (Liposomal)	20 mg	Injection	2	GPNA	11,520,00	12,624.00	9.60	12,072.00	–
Epirubicin Hydrochloride	10 mg	Injection	4	GPNA	720.00	1184.00	64.40	813.00	349.50
	50 mg	Injection	8	GPNA	3200.00	4400.00	37.50	4082	1621.70
Irinotecan	40 mg	Injection	4	GPNA	2604.60	14,495.40	456.50	3250.80	8960.30
	100 mg	Injection	5	GPNA	5250.00	36,241.90	590.30	7031.20	16,395.00
L-Asparaginase	5000 IU	Injection	2	1921.38	1504.40	1921.40	27.70	1712.90	–
	10,000 IU	Injection	2	GPNA	2330.70	2551.60	9.50	2441.10	–
Paclitaxel	100 mg	Injection	7	GPNA	3760.00	7600.00	102.10	6045.80	860.70
	260 mg	Injection	5	GPNA	10,432.00	17,600.00	68.70	15,360.00	4286.20
Vincristine	1 mg	Injection	2	89.39	80.50	89.40	11.00	84.90	–
Vinorelbine	10 mg	Injection	2	GPNA	5065.60	6374.20	25.80	5719.90	–
Anastrozole	1 mg	Tablet	5	GPNA	48.80	92.22	89.00	77.60	30.00
Tamoxifen	20 mg	Capsule	2	4.90	4.30	4.90	13.40	4.61	–
Letrozole	2.5 mg	Tablet	3	GPNA	33.30	57.60	73.10	46.00	–
Bevacizumab	100 mg	Injection	2	GPNA	43,200.00	51,811.70	19.90	47,505.80	–
Ceftuximab	100 mg	Injection	2	GPNA	31,000.00	32,355.20	4.40	31,677.60	–
Rituximab	500 mg	Injection	2	GPNA	12,756.80	12,757.20	0	12,757.00	–
	100 mg	Injection	2	GPNA	63,785.60	63,786.20	0	63,785.90	–
Trastuzumab	150 mg	Injection	2	GPNA	35,915.20	37,683.40	4.90	36,799.30	–
	440 mg	Injection	2	GPNA	97,039.50	99,270.10	2.30	98,154.80	–
Bortezomib	2 mg	Injection	5	GPNA	14,400.00	20,640.00	43.30	18,080.00	5640.00
Erlotinib	100 mg	Tablet	4	GPNA	533.30	1066.60	100.00	948.00	405.90
	150 mg	Tablet	5	GPNA	634.70	1599.80	152.10	1280.00	762.60
Geftinib	250 mg	Tablet	5	GPNA	272	567.40	108.60	368.40	206.40
Imatinib	100 mg	Tablet	5	154.74	112.60	154.70	37.40	135.10	36.40
	400 mg	Capsule	4	474.03	324.30	406.00	25.20	364.80	63.70

*GPNA* Government price not available, *NRs* Nepalese rupees, *IQR* Interquartile Range

Table 2 shows the maximum price variability (percentage) among various categories of anticancer drugs. Out of six categories of anticancer medicines, there were 240 anticancer products with different strengths and dosage form. Maximum price variability (percentage) was found in natural products (590.30%). There was variation in the price of all alkylating agents manufactured by different pharmaceutical companies. Within alkylating agents, temozolomide 100 mg capsule showed the maximum price variation (307.72%) whereas cyclophosphamide 500 mg injection showed the minimum price variation (3.33%). With respect to the antimetabolites agents, different brands of cytarabine 500 mg injection and gemcitabine 200 mg injection was available with no or minimal variation in price. However, pemetrexed 500 mg capsule showed the maximum price variation (134.01%). Within the natural products category, 590.32% variation in price was found in irinotecan 100 mg injection and minimum variation was found to be 9.58% in doxorubicin (liposomal) injection. Among hormonal therapy, three agents were available whose maximum price variation was found to be 200% at letrozole 2.5 mg tablet. The minimum price variation (13.88%) was found in the case of tamoxifen 20 mg capsule. Among monoclonal antibody, trastuzumab 440 mg injection showed the maximum price variation of 72.73%. With respect to miscellaneous agents, a different brand of bortezomib, erlotinib, gefitinib and imatinib were available. Bortezomib 2 mg injection showed the maximum price variation (240.87%) while imatinib 400 mg capsule showed the minimum price variation of 43.75%.

**Table 2:** Price variation among categories of anticancer agents

Category	N	Median	IQR	Minimum	Maximum	Minimum Variation Found	Maximum Variation Found
Alkylating agents	65	1920.00	3515.20	51.20	12,000.00	Cyclophosphamide 500 mg inj 3.33%	Temozolomide 100 mg 307.70%
Antimetabolites	48	6480.00	8729.54	187.36	48,000.00	Cytosine Arabinoside (Cytarabine) 500 mg Inj 0%	Pemetrexed 500 mg 134.00%
Natural products	77	5030.00	9017.60	80.48	36,241.92	Doxorubicin (liposomal) inj 9.58%	Irinotecan 100 mg 590.30%
Hormonal Therapy	10	53.20	44.32	4.32	92.22	Tamoxifen 20 mg capsule 13.88%	Letrozole 2.5 mg tablet 200.00%
Monoclonal antibody	12	40,441.69	32,108.32	12,756.80	99,270.12	Rituximab 100, 500 mg inj 0%	Trastuzumab 440 mg 72.73%.
Miscellaneous agents	28	550.40	1000.51	112.64	20,639.98	Imatinib 400 mg capsule 43.75%.	Bortezomib 2 mg injection 240.87%
**Total**	**240**						

Table 3 shows there was a significant difference in the mean MRP of the different anticancer medicine categories. Table 3 showed that there was a significant difference in the mean MRP of the alkylating agents with the antimetabolites (*p*-value 0.006) and the monoclonal antibody (*p*-value <.001). Antimetabolites, natural products, hormonal therapy all had significant mean differences in their MRPs with the monoclonal antibodies. (*p*-value <.001) and the monoclonal antibodies had a significant mean difference in the MRP with the miscellaneous agents. (*p*-value <.001).

**Table 3:** Category-wise comparison of MRP of anticancer agents

(I) Category	(J) Category	Mean Difference (I-J)	***p***-value
Alkylating agents	Antimetabolites	− 6528.71^*^	0.006
	Natural products	− 4397.18	0.102
	Hormonal Therapy	2734.06	1.000
	Monoclonal antibody	−45,661.74^*^	<.001
	Miscellaneous agents	− 850.56	1.000
Antimetabolites	Natural products	2131.52	1.000
	Hormonal Therapy	9262.77	0.086
	Monoclonal antibody	−39,133.03^*^	<.001
	Miscellaneous agents	5678.14	0.198
Natural products	Hormonal Therapy	7131.24	0.412
	Monoclonal antibody	−41,264.56^*^	<.001
	Miscellaneous agents	3546.62	1.000
Hormonal Therapy	Monoclonal antibody	−48,395.80^*^	<.001
	Miscellaneous agents	− 3584.62	1.000
Monoclonal antibody	Miscellaneous agents	44,811.18^*^	<.001

Table 4 shows a comparison of the market price of anticancer medicines available in two private hospital pharmacies of Nepal with government fixed price. GoN has fixed the MRP of 45 anticancer medicines (Available in Supplementary File 1). Out of 45 anticancer medicines, 16 anticancer medicines were not available in both the hospital pharmacies. Ten anti-cancer medicines were available with only one brand. The MRP of the available brand was compared with the price fixed by the government. The negative price variation was found 60.90% in vinblastine injection 10 mg, which is lower than the price set by the government. Similar negative variation was found in other anti-cancer medicines, where the available medicines were below or equal to the price set by the government.

**Table 4:** Comparison of the market price of anticancer medicines available in two private hospital pharmacies of Nepal with government fixed price

S.NO	Generic Name	Dosage	Strength	Government Price (NRs per unit)	No. of available brands	Min. Price (NRs per unit)	Max. Price (NRs per unit)	Price Variation (%)	Essential List
1	5 Flurouracil	injection/ml	250 mg/5 ml	3.68	0	NA	–	–	N
2	Actinomycin D	injection	0.5 mg	935.44	1	–	934.40	- 0.10	N
3	Alpha Interferon	injection	3 miu	1342.08	0	NA	–	–	N
4	Bleomycin	injection/ml	15 mg	1088.32	2	946.27	978.56	- 11.20	E
5	Busulphan	tablet	2 mg	6.03	0	NA	–	–	N
6	Carboplatin	injection	150 mg	1393.87	8	1184.00	1393.60	0	E
7	Carboplatin	injection	450 mg	4130.77	7	3476.80	4028.00	- 2.60	E
8	Chlorambucil	tablet	2 mg	79.89	0	NA	–	–	E
9	Cisplatin	injection	10 mg	149.87	5	100.00	115.20	- 30.10	E
10	Cisplatin	injection	50 mg	532.21	6	512.00	532.20	0	E
11	Cyclophosphamide	tablet	50 mg	6.62	0	NA	–	–	E
12	Cyclophosphamide	injection	500 mg	105.23	2	101.84	105.23	0	E
13	Cyclosporine	capsule	25 mg	43.71	0	NA	–	–	E
14	Cyclosporine	capsule	50 mg	85.25	0	NA	–	–	N
15	Cyclosporine	capsule	100 mg	187.78	0	NA	–	–	N
16	Cyclosporine	injection	100 mg/ml	209.60	0	NA	–	–	N
17	Cytosine Arabinoside	injection	100 mg	421.23	3	279.39	421.23	0	E
18	Cytosine Arabinoside	injection	500 mg	910.74	2	807.44	807.55	- 12.80	E
19	Cytosine Arabinoside	injection	1000 mg	2068.00	2	1604.80	2068.00	0	N
20	Dacarbazine	injection	500 mg	1817.98	2	1600.00	1729.00	5.10	E
21	Daunorubicin	injection	20 mg	592.18	2	592.00	592.17	0	E
22	Doxorubicin	injection	10 mg	328.00	5	264.00	328.00	0	E
23	Doxorubicin	injection	50 mg	1416.28	7	1280.00	1416.28	0	E
24	Etoposide	capsule	100 mg	92.40	1	–	88.40	- 4.50	E
25	Etoposide	injection	100 mg/5 ml	320.27	1	–	300.80	- 6.50	E
26	Flutamide	tablet	250 mg	15.22	0	NA	–	–	N
27	Gemcitabine	injection	200 mg	1706.93	8	1704.00	1706.90	0	N
28	Gemcitabine	injection	1000 mg	9980.00	10	7072.00	9980.00	0	N
29	Ifosfamide	injection	1 g/2 ml	609.26	1	–	609.24	0	E
30	Imatinib	tablet	100 mg	154.74	5	112.60	154.70	0	N
31	Imatinib	tablet	400 mg	474.03	4	324.30	466.20	- 1.70	N
32	L-Asparaginase	injection	500 iu	1921.38	2	1504.41	1703.60	- 12.80	N
33	Melphalan	tablet	2 mg	191.73	0	NA	–	–	E
34	Melphalan	tablet	5 mg	321.63	0	NA	–	–	E
35	Mercaptopurine	tablet	50 mg	15.78	0	NA	–	–	E
36	Methotrexate	injection/ml	50 mg/ml	58.62	1	–	58.62	0	N
37	Methotrexate	tablet	2.5 mg	8.34	1	–	8.33	- 0.10	E
38	Mitomycin C	injection	10 mg	702.35	1	–	702.35	0	E
39	Oxaliplatin	injection	50 mg	4035.86	7	3336.50	4035.86	0	N
40	Paclitaxel	injection	30 mg/5 ml	516.18	0	NA	–	–	N
41	Procarbazine	capsule	50 mg	55.89	0	NA	–	–	E
42	Tamoxifen	capsule	20 mg	4.90	2	4.32	4.90	0	E
43	Tamoxifen	capsule	10 mg	7.68	0	NA	–	–	N
44	Vinblastine	injection	10 mg	503.31	1	–	312.81	- 60.90	E
45	Vincristine	injection	1 mg/ml	89.40	2	80.40	89.40	0	E

*GPNA* Government price not available, *NRs* Nepalese rupees, *NA* Not available, *E* Listed in National list of essential medicines Nepal (fifth revision), *N* Not listed in National list of essential medicines Nepal (fifth revision), −: Negative Price variance

We also observed that all price of 27 anticancer medicines listed in the National list of essential medicines Nepal (fifth revision) (Available in Supplementary File 2) was regulated by the DDA. Seven medicines that were listed in the National list of essential medicines Nepal were not available in both hospital pharmacies. Nine medicines that are not listed in the National list of essential medicines Nepal were not available in both hospital pharmacies.

## Discussion

Nepal does not have a clear and transparent medicine pricing policy and even when policy exists for pricing, they are not implemented strictly. Usually, the cost of pharmaceutical products in Nepal is determined by the pharmaceutical industries based on their cost of production, marketing cost, and desired benefit. However, the practices of free pricing with cost-plus (pricing strategy of determining the selling price by adding a specific amount markup to that product’s cost price per unit) and the requirement of displaying the MRP are applied for all pharmaceutical products. Currently, the DDA asks international manufacturers’ authorized importers for comparative prices of medicines prior to issuing the marketing authorization [17] and there is provision for controlling prices of the drug as per the Drug Act 1978 [23]. However, the implementation has been weaker. Till now, the DDA has regulated the prices of only 117 pharmaceutical products; however, it does not include a large chunk of medicines that are used for chronic conditions and specialized diseases such as cancerand stroke [17].

The National list of essential medicines Nepal (fifth revision, first prepared in 1986) 2016 [24, 25], issued by the GoN has listed 38 anticancer medicines belonging to different categories of anticancer agents. Many of these 38 anticancer medicines on the National list of essential medicines Nepal were available in the medicines list that we investigated. However, seven anticancer medicines were unavailable which might be due to a regular shortage of medicines in Nepal [26].

To the best of our knowledge, this is the first study analyzing price variation among different brands of anticancer medicines available in the selected cancer hospitals of Nepal. This study shows considerable (up to 590.32%) variation in the price of anticancer medicines in Nepal. However, some brands of anticancer medicines such as actinomycin D, bleomycin, carboplatin, cisplatin, and cytosine arabinoside were available even at prices below the government fixed price which might be due to price-fixing and market competition. Kolasani et al. reported high-cost variation with hormonal drugs, flutamide 250 mg tablet i.e. 714.24% and lowest with targeted drug imatinib mesylate (100 mg; film-coated tablet) i.e. 5.56% [9]. Likewise, Adwal and Baghel’s study reported high-cost variation with alkylating agent carboplatin 150 mg injection i.e. 1100% and lowest with antimetabolite anticancer agent cytarabine 500 mg injection 6.56% [27]. Another study by Tadesse and Fang from Ethiopia shows that the lowest price generic anticancer medicines were sold 1.29 and 2.62 times the international reference price in the public and private sectors [28]. A study by Vogler S et al., where the investigator surveyed the prices of cancer drugs in high-income countries (16 European countries, Australia, and New Zealand) found that the price of new cancer drugs varies widely from 28 to 388% [8]. The huge variation in the price of anticancer medicines in our study might be due to differences in the production cost, price control policy not covering some of the medicines, prescribing by brand name and pharmaceutical companies influencing the prescriber for writing their brand using different strategies [29, 30].

The GoN has fixed the MRP of 117 commonly used medicines, including medicines for chronic diseases and 45 anticancer medicines (with different strengths and dosage forms) [31]. This step can help curb down the price of medicines by decreasing bonuses and other deals and improving access to essential medicines for common people. On the contrary, price regulation can sometimes inadvertently set medicines price at a level that might not be profitable for the seller and they may withdraw the product from the market [32]. Such cases have not been reported in Nepal; however, it provides ground for medicines sellers to ward off their competitors, especially when regulatory agencies are weaker due to poor governance and the sociopolitical problem of the Nepalese health system.

In developed countries such as US and Australia, the cost of anticancer treatment is funded through public and private health insurance plans along with no or some contribution from self-funding / out-of-pocket payments depending on the type of service received [33–35]. But, in Nepal, the government provides limited free basic medicines in public health sectors (health posts, primary health care centers and district hospitals) [19] and limited funding is available for the treatment of serious health issues such as cardiac surgery, renal transplant, dialysis, neurosurgery, and cancer. The Nepal government provides NRs 100,000 (Euro 877.19, USD 925.92) for cancer treatment [13]), sickle cell anemia, thalassemia and other life-threatening diseases [36]. However, the majority of the treatment cost is to be borne out-of-the-pocket by the families [13, 15, 16, 36]. In this context, huge difference in the price of same medicine (by a different manufacturer) as found in this study might affect patients’ expenditure on medicines, especially when the patient does not know about variation in prices for different manufacturers or when they are prescribed with a certain brand that is expensive. Creating a transparent pricing system and an efficient regulatory ecosystem might help curb some of the medicines pricing related issues such as high price variation.

At the national policy level, different strategies such as cost-plus pricing regulation, external reference pricing, promotion of generics, and tax exemption policy, are available to reduce the prices of medicines, including anticancer medicine in developing countries like Nepal [37, 38]. However, at an international level, the recent trend has been to move away from cost-plus to a value-based pricing system [39]. The same strategy is used by the DDA to control the price of 117 pharmaceutical products based on the concept of mean, median and quartile prices of common brands and external reference pricing [31]. Global best practices show that transparent pricing policies, price strategies based on usage data and better regulation and monitoring are required to curb down both high prices and a huge variation in prices of anticancer medicines [38, 40].

At the institutional level, the Drug and Therapeutics Committee (DTC) of the hospital can play a role in selecting safe, efficacious and affordable medicines. However, most of the Nepalese hospitals lack a DTC and some of them are not functioning properly [26]. The hospitals included in this study have a DTC but due to lack of proper manpower and lack of time from doctors, DTC was not functioning properly. Both the hospitals in our study did not have hospital formulary that could have checked and curbed any kind of malpractice and helped promote the quality use of relatively cost-effective anticancer medicines. A study by Khatiwoda et al. reported that medical cost contributed most to the direct cost, out-of-the-pocket payment was the dominant mechanism and most of the patients faced financial hardship due to unaffordable medical cost [13]. The study also reported the financial subsidy to be insufficient and overall cost (medical, non-medical and direct cost) to be significantly linked with socioeconomic status, age, cancer type, and treatment. A combined approach including better healthcare financing, resource allocation based on socioeconomic status and other related factor and healthcare delivery system that promotes the quality use of anticancer medicines is required.

Hospital pharmacists can help promote the usage of appropriate medicines by carrying out cost-effectiveness and cost-benefit analysis of anticancer medicines [41]. Evidence suggests that clinical pharmacists’ interventions during cancer therapy can help in the cost-effectiveness approach and adopt advanced cancer treatment with optimum and affordable cost [42–44]. The physicians and patients themselves too can play some role in selecting affordable anticancer medicine.

The variation in price may also be reduced if the Nepalese domestic pharmaceutical companies manufactured anticancer medicines. The GoN should develop policies that promote a tie-up of the Nepalese manufacturing company with international pharmaceutical companies so that they can produce some anticancer medicines for the Nepalese Market. As of now, price fixation up to a tolerable limit, transparent pricing policy and pricing based on anticancer medicine usage data would be better strategies to improve the overall access to anticancer medicines for Nepalese patients.

## Limitations

Some anticancer medicines available in the Nepalese market might be missed as this study only compared the price of different anticancer medicines available at two private hospital pharmacies of a cancer hospital in Kathmandu Valley. Both hospitals lack a hospital-based cancer registry, due to this exact proportion of cancer patients in these two hospitals couldn’t be found out. The government hospitals of Kathmandu valley where most patients get treated were not included in this study as the hospital pharmacy is not run by the hospital itself and it was another limitation of this study. Inclusion of all or most of the anticancer medicines and prices from a government hospital would have provided a better understanding of the variation in prices of anticancer medicine and the findings would have been more generalizable. Non-inclusion of the different combinations of anticancer medicines was another drawback of this study. Procurement prices of anticancer medicines could not be examined due to the privacy policy of the procurement committee of the hospitals. The inclusion of procurement price would have given an estimate of the average markups by the pharmacy.

## Conclusion

Our study exhibits considerable variation in the price of different brands and among different categories of the anticancer medicines available in the two cancer hospitals of Nepal. The currently adopted price control policy by the GoN was found to be effective in reducing the price as well as the variation in such price of medicine manufactured by different pharmaceutical companies and, thus, this policy, should be extended to other medications used for the management and treatment of NCDs. Generic prescribing (maintaining the quality, safety, and efficacy), external reference pricing, tax exemption and stringent assessment of the implementation of current policies would aid in reversing the augmented pattern of price variation of anticancer medicines and by providing cost-competitive options, provide much-needed relief to the patients.

## Supplementary information

**Additional file 1.**

**Additional file 2.**

## Data Availability

The datasets used and/or analyzed during the current study are available from the corresponding author on reasonable request.

## Abbreviations

DDA: Department of Drug Administration
DTC: Drug and Therapeutic Committee
GoN: Government of Nepal
MRP: Maximum Retail Price
NCDs: Non-communicable diseases
