# Supplementary material for: Price variation among different brands of anticancer medicines available in hospital pharmacies of Nepal
Source: J Pharm Policy Pract. 2020 Apr 2;13:6. doi: 10.1186/s40545-020-0203-0 (PMC7118972; doi:10.1186/s40545-020-0203-0)
Supplement: Supplementary file 1 — Additional file 1. [file 40545_2020_203_MOESM1_ESM.pdf]

Nepal Gazette

Published by Nepal Government

Part 65) Kathmandu August 3, 2015 A.D. (Number 12

Notice 2

Pursuant to decision of Ministerial Council of Nepal Government dated July 06, 2015 A.D. using the right according to section 26 of the Drug Act, 1978 following maximum retail price has been fixed as mentioned in the schedule 1 & 2, so that this notice has been published for public information.

Schedule 1

Fixed maximum retail price of 18 most commonly used products

S.N	Medicine	Unit	Price/Unit (NRs.)
1	Cefpodoxime 200 mg	Tablet/Capsule	25.00
2	Amoxycillin 500 mg	Tablet/Capsule	8.00
3	Amoxycillin 250 mg	Tablet/Capsule	5.00
4	Azithromycin 500 mg	Tablet/Capsule	30.00
5	Azithromycin 250 mg	Tablet/Capsule	17.45
6	Cefixime 200 mg	Tablet/Capsule	20.00
7	Ceftriaxone 1g Inj	Vial/Ampoule	90.00
8	Ceftriaxone 500 mg Inj	Vial/Ampoule	60.00
9	Ciprofloxacin 500 mg	Tablet/Capsule	8.00
10	Fluconazole 150 mg	Tablet/Capsule	24.00
11	Levonorgestrol 150 mcg tab or 2 tablets of levonorgestrol 75 mg	Tablet/Capsule	80.00
12	Nimesulide 100 mg	Tablet/Capsule	3.00
13	Ofloxacin 400 mg	Tablet/Capsule	8.00
14	Omeprazole 20 mg	Tablet/Capsule	4.00
15	Pantoprazole 40 mg	Tablet/Capsule	8.00
16	Tinidazole 500 mg	Tablet/Capsule	5.00
17	Levofloxacin 750 mg	Tablet/Capsule	15.00
18.	Combination of Paracetamol with Chlorpheniramine, and Phenylepherine or pseudoephedrine (Anticold Tablets/Capsules)	Tablet/Capsule	3.00

NOTARY PUBLIC नोटरी पब्लिक Name: Rameshwor Pant Regd. No. 3277 Expiry Date: July09, 2023 AD Date: July08, 2018 AD District: Lalitpur नेपाल नोटरी पब्लिक परिषद् NEPAL NOTARY PUBLIC COUNCIL

"The Translation Copy is True and Verified"
Signature:
Name: Rameshwor Pant
Date: 14/01/2020
Certificate Number of the Notary Public: 3277
Date of Expiry of Certificate: July09, 2023 AD
Seal of the Notary Public

Schedule 2

Fixed maximum retail price of 78 products to be used in treatment of cancer including long term diseases

S.N	Medicine	Unit	Price/Unit (NRs.)
1	Amlodipine 2.5 mg	Tablet/Capsule	2.30
2	Amlodipinne 5 mg	Tablet/Capsule	5.00
3	Amlodipine 10 mg	Tablet/Capsule	8.00
4	Losartan 25 mg	Tablet/Capsule	4.41
5	Losartan 50 mg	Tablet/Capsule	7.60
6	Atorvastatin 5 mg	Tablet/Capsule	6.75
7	Atorvastatin 10 mg	Tablet/Capsule	10.78
8	Atorvastatin 20 mg	Tablet/Capsule	20.60
9	Enalapril 2.5 mg	Tablet/Capsule	2.30
10	Enalapril 5 mg	Tablet/Capsule	4.00
11	Enalapril 10 mg	Tablet/Capsule	7.00
12	Metoprolol 12.5 mg	Tablet/Capsule	2.90
13	Metoprolol 25 mg	Tablet/Capsule	4.00
14	Metoprolol 50 mg	Tablet/Capsule	5.30
15	Metformin 500 mg	Tablet/Capsule	2.00
16	Metformin 1000 mg tab	Tablet/Capsule	6.00
17	Glimepride 1 mg	Tablet/Capsule	5.30
18	Glimepride 2 mg	Tablet/Capsule	8.60
19	Glimepride 3 mg	Tablet/Capsule	11.20
20	Pioglitazone 15 mg tab	Tablet/Capsule	6.50
21	Pioglitazone 30 mg tab	Tablet/Capsule	10.80
22	Amityptalline 10 mg	Tablet/Capsule	2.40
23	Amityptalline 25 mg	Tablet/Capsule	3.80
24	Imipramine 25 mg tab	Tablet/Capsule	1.32
25	Imipramine 75 mg	Tablet/Capsule	3.00
26	Lorazepam 1 mg	Tablet/Capsule	2.00
27	Lorazepam 2 mg	Tablet/Capsule	2.80
28	Alprazolam 0.25mg tab	Tablet/Capsule	1.70
29	Alprazolam 0. 5mg	Tablet/Capsule	3.40
30	Carboplatin 150 mg	Injection	1,393.87
31	Carboplatin 450 mg	Injection	4,130.77
32	Chlorabucil 2 mg	Tablet	79.89
33	Cisplatin 10 mg	Injection	149.87
34	Cisplatin 50 mg	Injection	532.21
35	Cyclophosphamaide 50 mg	Tablet	6.62
36	Cyclophosphamaide 500 mg	Injection	105.23
37	Cytosine Arabinoside 100 mg /vial	Injection	421.23
38	Cytosine Arabinoside 500 mg /vial	Injection	910.74

NOTARY PUBLIC नोटरी पब्लिक
Name: Rameshwor Pant
Regd. No. 3277
Expiry Date: July09, 2023 AD
Date: July09, 2018 AD
District: Lalitpur
NEPAL NOTARY PUBLIC COUNCIL नेपाल नोटरी पब्लिक परिषद्

"The Translation Copy is True and Verified"
Signature:
Name: Rameshwor Pant
Date: 14/01/2020
Certificate Number of the Notary Public: 3277
Date of Expiry of Certificate: July09, 2023 AD
Seal of the Notary Public

39	Cytosine Arabinoside 1000 mg /vial	Injection	2,068.00
40	Dacarbazine 500 mg	Injection	1,817.98
41	Danazol 50 mg	Capsule	16.54
42	Danazol 100 mg	Capsule	35.90
43	Daunorubicin 20 mg	Injection	592.18
44	Doxorubicin 10 mg	Injection	328.00
45	Doxorubicin 50 mg	Injection	1,416.28
46	Etoposide 100 mg	Capsule	92.40
47	Etoposide 100 mg/5 ml	Injection	320.27
48	Flutamide 250 mg	Tablet	15.22
49	5 flurouracil 250 mg/ 5 ml	Injection / ml	3.68
50	Gemcitabin 200 mg	Injection	1,706.93
51	Gemcitabin 1000 mg	Injection	9,980.00
52	L- asparginase 500 KU	Injection	1,921.38
53	Mercaptopurine 50 mg	Tablet	15.78
54	Imatinib 100	Tablet	154.74
55	Imatinib 400	Tablet	474.03
56	Ifosamide 1 gm/2ml	Injection	609.26
57	Methotraxate 50 mg/ml	Injection / ml	58.62
58	Methtraxate 2.5 mg	Tablet	8.34
59	Mensa 200 mg	Injection / ml	43.41
60	Mitomycin c 10 mg	Injection	702.35
61	Oxaliplatin 50 mg /vial	Injection	4,035.86
62	Paclitaxel 30 mg/5 ml	Injection	516.18
63	Procarbazine 50 mg	Capsule	55.89
64	Tamoxifen 10 mg	Tablet	7.68
65	Tamoxifen 20 mg	Tablet	4.90
66	Vinblastin 10 mg/ pack	Injection	503.31
67	Vincristin 1 mg / ml	Injection	89.39
68	Melphalan 2 mg	Injection	191.73
69	Melphalan 5mg	Tablet	321.63
70	Azathioprine 50 mg	Tablet	17.47
71	Cyclosporine 25 mg	Capsule	43.71
72	Cyclosporine 50 mg	Capsule	85.25
73	Cyclosporine 100 mg	Capsule	187.78
74	Cyclosporine 100 mg/ml	Injection / ml	209.60
75	Actinomycin D 0.5 mg	Injection	935.44
76	Alpha Interferon 3 MIU	Injection	1,342.08
77	Bleomycin 15 mg	Injection / ml	1,088.32
78	Busulphan 2 mg	Tablet	6.03

By order,
Raghuram Bista
Secretary for Nepal Government

NOTARY PUBLIC
नोटरी पब्लिक
Name: Rameshwor Pant
Regd. No. 3277
Expiry Date: July09, 2023 AD
Date: July08, 2018 AD
District: Lalitpur
नेपाल नोटरी पब्लिक परिषद्
NEPAL NOTARY PUBLIC COUNCIL

"The Translation Copy is True and Verified"
Signature:
Name: Rameshwor Pant
Date: 14/01/2020
Certificate Number of the Notary Public: 3277
Date of Expiry of Certificate: July09, 2023 AD
Seal of the Notary Public
