# Supplementary material for: Price variation among different brands of anticancer medicines available in hospital pharmacies of Nepal
Source: J Pharm Policy Pract. 2020 Apr 2;13:6. doi: 10.1186/s40545-020-0203-0 (PMC7118972; doi:10.1186/s40545-020-0203-0)
Supplement: Supplementary file 2 — Additional file 2. [file 40545_2020_203_MOESM2_ESM.pdf]

NATIONAL LIST OF ESSENTIAL MEDICINES NEPAL (FIFTH REVISION) 2016

GOVERNMENT OF NEPAL
MINISTRY OF HEALTH
DEPARTMENT OF DRUG ADMINISTRATION

	Miltefosine	capsule, 50 mg
	Amphotericin B	powder for injection, 50 mg in vial (as deoxycholate or liposomal)
	Sodium stibogluconate	injection, 100mg/ml
**6.5.3**	**Antimalarial Medicines**	
	Artemether*	oily injection, 80 mg/ml in 1-ml ampoule.
	** For use in the management of severe malaria.*	
	Artemether+lumefantrine*	tablet, 20 mg+ 120 mg
	** Not recommended in the first trimester of pregnancy or in children below 5 kg.*	
	Artesunate	injection, ampoules, containing 60 mg anhydrous artesunic acid with a separate ampoule of 5% sodium bicarbonate solution; tablet 50 mg
	(To be used in combination with Sulfadoxine + Pyrimethamine.)	
	Chloroquine	tablet, 150 mg base (as phosphate or sulfate); oral liquid, 50 mg / 5ml (as phosphate or sulfate); injection, 40mg /ml in 5- ml ampoule (as phosphate, sulfate or hydrochloride)
	Primaquine	tablet, 7.5 mg, 15 mg (as diphosphate)
	Sulfadoxine + Pyrimethamine	tablet, 500 mg + 25 mg
	(To be used only in combination with Artesunate.)	
	Quinine	tablet, 300 mg (as bisulfate or sulfate); injection, 300 mg (as dihydrochloride)/ ml in 2-ml ampoule.
**7**	**Antimigraine Medicines**	
**7.1**	**For Treatment of Acute Attack**	
	Paracetamol	tablet, 500 mg
**7.2**	**For Prophylaxis**	
	Propranolol	tablet 20 mg, 40 mg (hydrochloride)
**8**	**Antineoplastic, Immunosuppressives and Medicines Used in Palliative Care**	
**8.1**	**Immunosuppressive Medicines**	
	Cyclosporin	capsule 25 mg
**8.2**	**Cytotoxic Medicines**	
	Calcium folinate (Calcium leucovorin)	tablet, 15 mg
	Chlorambucil	tablet, 2 mg, 5 mg

	Cisplatin		powder for injection, 10 mg, 50 mg in vial.
	Cyclophosphamide		tablet, 25 mg; powder for injection, 200 mg, 500 mg, 1 g in vial
	Cytarabine		injection 100 mg, 500 mg in vial
	Dacarbazine		powder for injection, 100 mg in vial
	Dactinomycin		powder for injection, 500 mcg in vial
	Daunorubicin		powder for injection 20 mg (as hydrochloride) in vial
	Doxorubicin		powder for injection, 10 mg, 50 mg in vial
	Epirubicin		injection, 10 mg, 50 mg (hydrochloride) in vial
	Etoposide		tablet, 100 mg, injection 20 mg/ml in 5-ml ampoule
	Fluorouracil		injection 50 mg/ml in 5-ml, 10-ml ampoule
	Hydroxy urea		capsule 500 mg
	Ifosfamide + Mesna		injection, 1g + 200 mg, in vial
	Lomustine		capsule, 40 mg
	Melphalan		tablet, 2 mg, 5 mg; powder for injection, 50mg in vial
	Mercaptopurine		tablet, 50 mg
	Methotrexate		tablet, 2.5/5/10 mg (as sodium salt); powder for injection 15 mg, 50 mg (as sodium salt) in vial
	Mitomycin		powder for injection, 2mg, 10mg, 20mg in vial
	Mitoxantrone		injection, 2 mg/ml in 10ml ampoule
	Procarbazine		capsule 50 mg (as hydrochloride)
	Vinblastine		powder for injection 10 mg (sulfate) in vial
	Vincristine		powder for injection, 1 mg (sulfate) in vial
		Bleomycin	powder for injection, 15mg (as sulfate) in vial
		Carboplatin	injection 150 mg, 450 mg in vial
**8.3**	**Hormones and Antihormones**		
	Hydrocortisone		powder for injection, 100 mg (as sodium succinate) in vial
	Tamoxifen		tablet, 20 mg (as citrate)
		Bicalitumide	tablet or capsule 50 mg
		Dexamethasone	Dexamethasone phosphate 4 mg /ml (as sodium

		salt) in 2-ml ampoule
	Prednisolone	tablet, 5 mg, 10 mg, 20 mg
**8.4**	**Miscellaneous**	
	Granulocyte Colony Stimulating Factor (GCSF)	injection, 30 million unit in vial
	Interferon	injection, 5 million units/ml in vial
	L-Asparaginase	injection, 5 000 IU, 10 000 IU in vial
	Ondansetron	injection, 2 mg/ml (as hydrochloride) in 2-ml, 4-ml vial; tablet 2mg, 4mg (as hydrochloride)
**9**	**Antiparkinsonism Medicines**	
	Levodopa + Carbidopa	tablet, 100 mg + 10 mg, 250 mg + 25 mg
	Trihexyphenidyl (benzhexol)	tablet, 2 mg (hydrochloride)
**10**	**Medicines Affecting the Blood**	
**10.1**	**Antianaemia Medicines**	
	Ferrous sulfate*	tablet, equivalent to 60 mg iron; oral liquid, equivalent to 25 mg iron/ ml
	Ferrous sulfate*+Folic acid	tablet, equivalent to 60 mg Iron+ 400 mcg Folic acid, oral drop.
	**Ferrous fumarate may be used*	
	Folic acid	tablet, 5mg
	Iron Dextran	injection, equivalent to 50 mg iron/ml in 2-ml ampoule
**10.2**	**Medicines Affecting Coagulation**	
	Enoxaparin	injection 30mg/0.3ml, 60mg/0.6ml, 80mg/0.8ml
	Heparin sodium	injection, 1000 IU/ml, 5000 IU/ml,20000IU/ml in 1-ml ampoule
	Phytomenadione	injection, 10mg /ml in 5-ml ampoule; tablet 10 mg
	Protamine sulfate	injection, 10mg/ml in 5-ml ampoule
	Warfarin	tablet, 1 mg, 2 mg, 5 mg (sodium salt)
	Acenocoumarol	tablet, 1mg
**11**	**Blood Products and Plasma Substitutes**	
**11.1**	**Plasma Substitutes**	
	Albumin, human	injectable solution, 20 or 25%
	Polygeline	injectable solution, 3.5%
